# Structural and thermoelectric properties of TMGa_3_ (TM = Fe, Co) thin films

**DOI:** 10.3762/bjnano.4.54

**Published:** 2013-07-31

**Authors:** Sebastian Schnurr, Ulf Wiedwald, Paul Ziemann, Valeriy Y Verchenko, Andrei V Shevelkov

**Affiliations:** 1 Institute of Solid State Physics, Ulm University, D-89081 Ulm, Germany; 2 Department of Chemistry, Lomonosov Moscow State University, Moscow 119991, Russia

**Keywords:** amorphous metal films, energy related, intermetallic compounds, nanomaterials, Seebeck coefficient, thermoelectric properties, thin metal films

## Abstract

Based on chemically synthesized powders of FeGa_3_, CoGa_3_, as well as of a Fe_0.75_Co_0.25_Ga_3_ solid solution, thin films (typical thickness 40 nm) were fabricated by flash evaporation onto various substrates held at ambient temperature. In this way, the chemical composition of the powders could be transferred one-to-one to the films as demonstrated by Rutherford backscattering experiments. The relatively low deposition temperature necessary for conserving the composition leads, however, to ‘X-ray amorphous’ film structures with immediate consequences on their transport properties: A practically temperature-independent electrical resistivity of ρ = 200 μΩ·cm for CoGa_3_ and an electrical resistivity of about 600 μΩ·cm with a small negative temperature dependence for FeGa_3_. The observed values and temperature dependencies are typical of high-resistivity metallic glasses. This is especially surprising in the case of FeGa_3_, which as crystalline bulk material exhibits a semiconducting behavior, though with a small gap of 0.3 eV. Also the thermoelectric performance complies with that of metallic glasses: Small negative Seebeck coefficients of the order of −6 μV/K at 300 K with almost linear temperature dependence in the range 10 K ≤ *T* ≤ 300 K.

## Introduction

Intermetallic compounds usually behave as metals. In some cases, however, when a compound contains both, d- and p-block metals, semiconducting behavior may emerge. The number of such semiconducting intermetallic compounds is quite limited. For instance, RuAl_2_ and RuGa_2_ with TiSi_2_ structure type [1], some Heusler alloys such as Fe_2_VAl [2], and several intermetallics of FeGa_3_ structure type [3–4] are known to be semiconductors, at least as bulk samples. The formation of the band gap in the isostructural compounds FeGa_3_, RuGa_3_ and RuIn_3_ originates from the hybridization of the narrow d-bands of the transition metal (TM) with rather broad p-bands of the group-III elements. In particular, such a hybridization also produces sharp features in the electronic density of states (DOS) close to the Fermi level, which are expected to be quite beneficial for an enhanced thermoelectric response [5–6]; large Seebeck coefficients of −350 μV/K [7] or even −563 μV/K [8] at room temperature were reported for bulk FeGa_3_.

Recently, we found the existence of an unlimited solid solution between the isostructural intermetallics FeGa_3_ and CoGa_3_ [9]. With an increasing cobalt content in the Fe_1−_*_x_*Co*_x_*Ga_3_ solid solution, the Fermi level shifts up to the conduction band and crosses peaks of high electronic density of states, ultimately leading to metallic and non-magnetic properties for CoGa_3_. Thus, the composition of the solid solution *x* was found to be a tool to control the number of electronic states at the Fermi level *N*(*E*_F_) when the variation of *N*(*E*_F_) for different *x* was established from the results of band structure calculations and the nuclear quadrupole resonance (NQR) investigations of the nuclear spin–lattice relaxation rate. In line with these results, the Fe_1−_*_x_*Co*_x_*Ga_3_ solid solution was found to behave as a metal for *x* > 0.025. For smaller values of *x* the system remains non-metallic, while the density of states at the Fermi level for 0 < *x* ≤ 0.025 increases drastically in comparison with pure FeGa_3_. Such a sharp feature of *N*(*E*_F_) should lead to an appreciable thermoelectric performance, which can be tuned by accurate adjustment of the Co content.

Thus, having Fe_1−_*_x_*Co*_x_*Ga_3_ solid solutions with tunable electronic properties available, the prospect of applications related to miniaturized sensors or generators of electrical energy naturally motivates to try and prepare corresponding thin films as well. This aim, however, immediately poses the question as to the most appropriate preparational method. Starting in the present work with hot-pressed pellets of FeGa_3_ and CoGa_3_, as well as of an Fe_0.75_Co_0.25_Ga_3_ solid solution, one faces the main problem of picking a deposition technique which conserves these starting chemical compositions. Previous experience suggested applying pulsed laser deposition (PLD) for that purpose. However, it turned out that the pressed targets were not sufficiently stable but rather mechanically disintegrated during the ablation process. Thus, alternatively, thermal grain-by-grain evaporation from a powder source was applied leading to an averaging of the chemical composition over the thickness of the resulting films. In this way, stoichiometry changes due to fractional evaporation can be avoided as will be discussed below. Fractional evaporation and film disintegration is also a critical topic in the context of the preparation at elevated substrate temperatures or subsequent sample annealing in order to improve film crystallinity. In the present study with its emphasis on thermoelectric properties of the (TM)Ga_3_ films, the related figure of merit [10] ZT = *S*^2^σ*T*/λ (*S*: Seebeck coefficient, σ: electrical conductivity, λ: thermal conductivity, *T*: Kelvin temperature) indicates that low thermal conductivities may be of advantage in combination with reasonable high electrical conductivities. While the Seebeck coefficient is mostly dominated by asymmetric features of the electronic density of states *N*(*E*) around *E*_F_, σ and λ are influenced by both, electronic properties like *N*(*E*_F_) and the crystalline disorder effecting the corresponding transport mean-free-paths. As a consequence, in the present work focus is put on strongly disordered (TM)Ga_3_ films relaxing the above mentioned fractional evaporation problem at elevated temperatures. Indeed, all (TM)Ga_3_ films were evaporated onto substrates held at room temperature without subsequent annealing delivering nanocrystalline or even amorphous samples.

## Experimental

### Synthesis of bulk specimens

Powders of iron (Acros Organics, 99%) and cobalt (Alfa Aesar, 99.8%), and gallium rods (Aldrich, 99.999%) were used as received. Three specimens with chemical compositions FeGa_3_, Fe_0.75_Co_0.25_Ga_3_, and CoGa_3_ were prepared by a standard ampoule technique. For that, the starting materials with total mass of 4 g in each case were sealed in quartz ampoules under a vacuum of 10^−2^ torr. Ampoules were annealed in a programmable furnace at 500 °C for seven days. Thereafter, the obtained powders were thoroughly ground in an agate mortar, sealed in quartz ampoules and annealed in a furnace at 600 °C for another seven days. The phase composition of the specimens was analyzed through a standard X-ray technique using a Stoe STADI-IP diffractometer with Cu Kα_1_ radiation (Ge monochromator, λ_Cu_ = 1.540598 Å). In all cases powder diffraction patterns confirmed that single phase specimens were obtained (not shown). Calculated lattice parameters for the FeGa_3_, Fe_0.75_Co_0.25_Ga_3_, and CoGa_3_ are in good agreement with previously reported values [8]. Resulting powders were pressed into cylindrical pellets with a diameter of 10 mm and a height of ca. 5–6 mm. These pellets and powders served as the starting materials for the thin-film preparation.

### Preparation of thin films

The films were prepared by flash evaporation [11] of the corresponding FeGa_3_, Fe_0.75_Co_0.25_Ga_3_, or CoGa_3_ powder. For that purpose, a rotating tube (inner diameter 3.5 mm) with an internal thread transports the powder towards its end, where the powder falls grain-by-grain onto an electrically heated tungsten boat and evaporates. Each grain (typical diameter 10 μm) contributes significantly less than a monolayer to the growing film. Due to the statistically varying composition of the grains, the resulting film stoichiometry is averaged over the film thickness with the mean value corresponding to the composition of the starting powder. Film thicknesses in the range of 30–40 nm were realized at small rates of typically 1 nm/min as indicated by a quartz crystal monitor at a background pressure of 10^−8^ mbar with a cooling shield filled with liquid N_2_. For the lateral patterning of the films evaporation was performed through masks in contact with the substrates (c-cut sapphire or glass) held at ambient temperature in all cases. In this way, film stripes of 500 μm width and 1.6 mm length were obtained. In the case of four-point resistance measurements performed within a ^4^He-cryostat (in the range from 7 to 300 K by applying a current of 10 μA), the films were deposited on previously prepared gold contacts.

### Structural and compositional characterization of thin films

To extract structural information of the thin films deposited onto sapphire substrates, X-ray diffraction measurements were performed with a Panalytical X'Pert diffractometer (Cu Kα) equipped with a silicon-based position-sensitive X'Celerator detector. Information about the chemical composition of the (TM)Ga_3_ films was obtained by Rutherford backscattering spectroscopy (RBS) with 700 keV He^2+^ ions backscattered by 170° from samples deposited on silicon substrates. Simulating the experimental RBS spectra by the freely accessible software RUMP [12] delivers both, the chemical composition and the thickness of the films. Surface-roughness data of the (TM)Ga_3_ films were obtained by applying height profilometry (Veeco Dektak 150) and averaging along 200 μm long traces (needle curvature 2.5 μm, contact force 50 mN).

### Determination of thin film Seebeck coefficients

To determine the temperature-dependent Seebeck coefficients *S*(*T*) of (TM)Ga_3_ films, in a first step the films were complemented by strips of Pb to form (TM)Ga_3_/Pb-thermocouples arranged on a thin (140 μm) glass substrate. Since *S*(*T*) values for Pb are well documented in the literature [13], the corresponding values for (TM)Ga_3_ films can be extracted from such thermocouples. The glass substrate is bridging the gap between the two parts of a split Cu sample holder, each half of which is equipped with a heater and thermometer allowing the temperature to be controlled independently. Thus, while ramping up the temperature of one half, the temperature of the other one is kept constant. When the resulting temperature difference Δ*T* reaches its maximum value Δ*T*_max_, heating is reversed until, after crossing Δ*T* = 0, the opposite maximum −Δ*T*_max_ is obtained. By periodically repeating this cycle, the average temperature <*T*> linearly increases while Δ*T* exhibits a sawtooth-like behavior, which is closely followed by the corresponding sawtooth-curve for the thermoelectric voltage signal Δ*U*. The slope Δ*U*/Δ*T* then delivers the Seebeck coefficient *S*(<*T*>) assigned to the average temperature. Performing these measurements within a ^4^He cryostat allows the determination of *S*(*T*) values in the temperature range between 7 K and 300 K. More experimental details about the above procedure can be found in [14].

## Results and Discussion

**Figure 1:**
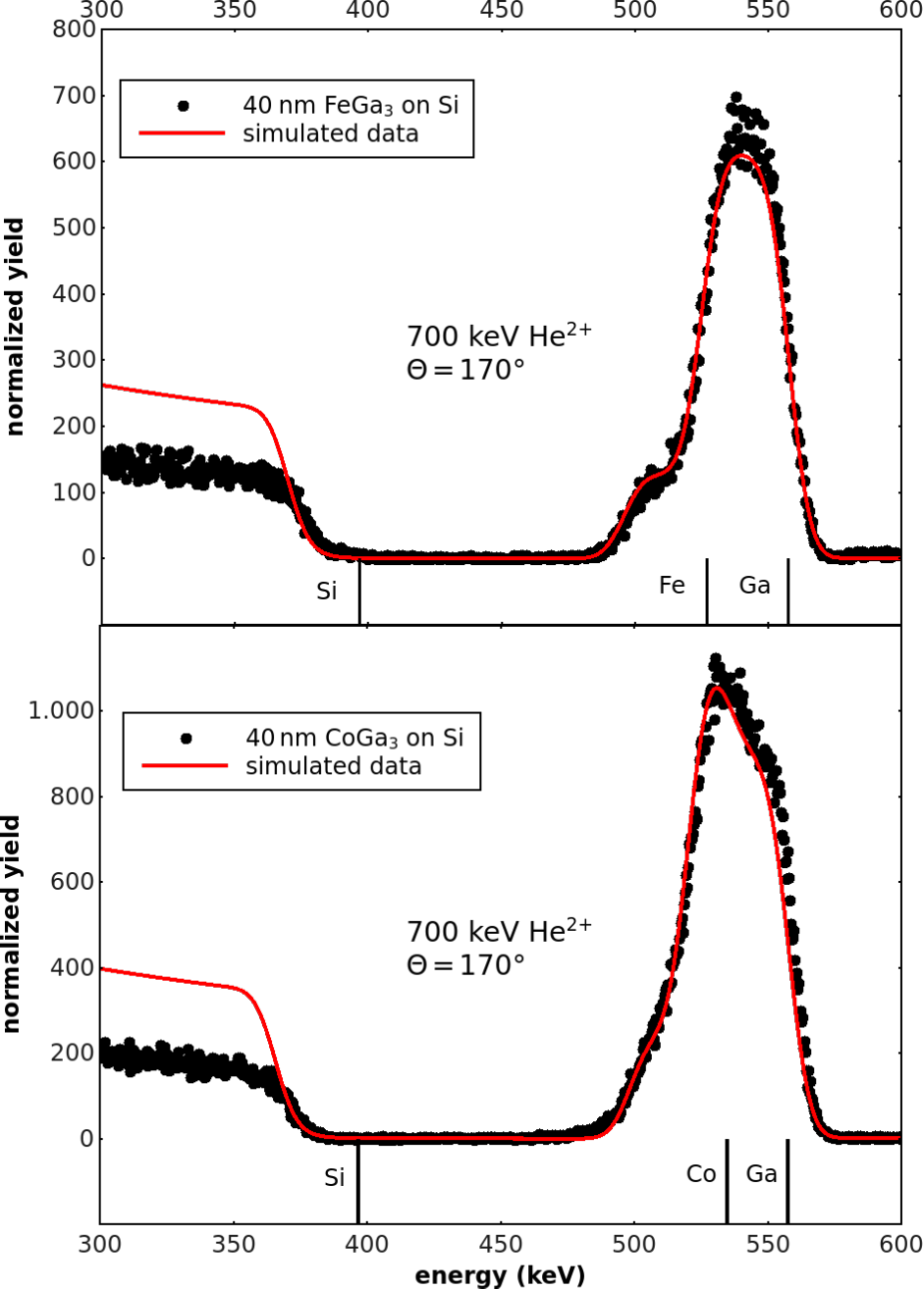
RBS spectra of FeGa_3_ (a) and CoGa_3_ (b) films deposited onto Si substrates. RUMP simulations (solid lines) deliver a composition of FeGa_3.2_ with a thickness of 40 nm and a composition of CoGa_3_ with a thickness of 43 nm. Backscattering energies of Co, Ga and Si at the sample surface are indicated by vertical marks. The displacement of the Si edge of the substrate toward lower backscattering energies is caused by the film thickness.

The first aim was to confirm the expectation that flash evaporation of powders consisting of grains with chemical compositions statistically fluctuating around an average value leads to thin films with a stoichiometry reflecting this average. For this purpose RBS experiments were performed and two examples of FeGa_3_ (42 nm) and CoGa_3_ (47 nm) films on Si substrates, respectively, are presented in Figure 1. The film thicknesses given in brackets were determined by an in situ quartz crystal balance during evaporation. The experimental data in Figure 1a and Figure 1b are supplemented by RUMP simulations indicating a composition FeGa_3.2_ with a film thickness of 40 nm and a composition CoGa_3_ with a film thickness of 43 nm. Given the typical RBS accuracy of 10%, in both cases the compositions are close to the expected ones of the starting material. Similarly, the thicknesses agree with those obtained from the quartz balance within an error of 9%. Thus, the RBS data confirm that flash evaporation of powders is an appropriate method to fabricate thin films reflecting closely the average chemical composition of the starting material. On the other hand, since Fe and Co are neighbors in the periodic table of the elements, their scattering contrast is too small to allow their thorough distinction in RBS. Thus, for Fe_1−_*_x_*Co*_x_*Ga_3_ solid solutions a determination of *x* by RBS was not possible.

Next, films prepared under identical conditions as those characterized by RBS were analyzed by XRD. Quite surprisingly, neither for FeGa_3_ nor CoGa_3_ any indication of Bragg peaks could be detected. Even the careful comparison of the film spectra to data of blank sapphire substrates tilted by 2° (to suppress the Bragg peaks of the single crystal) in order to provide a reference background did not reveal any significant differences. Thus, one can conclude that the film structure either is nanocrystalline with an average grain size below 4 nm or it is even amorphous. The conjecture of extremely small grains is supported by scanning electron microscopy (SEM) images taken with a high resolution Hitachi S5200 system (30 keV). Here, for all films completely featureless images were obtained suggesting flat amorphous or nanocrystalline samples with grains below the lateral SEM resolution of about 5 nm. Flatness could be corroborated by stylus measurements revealing a typical RMS averaged film roughness of 0.5 nm.

**Figure 2:**
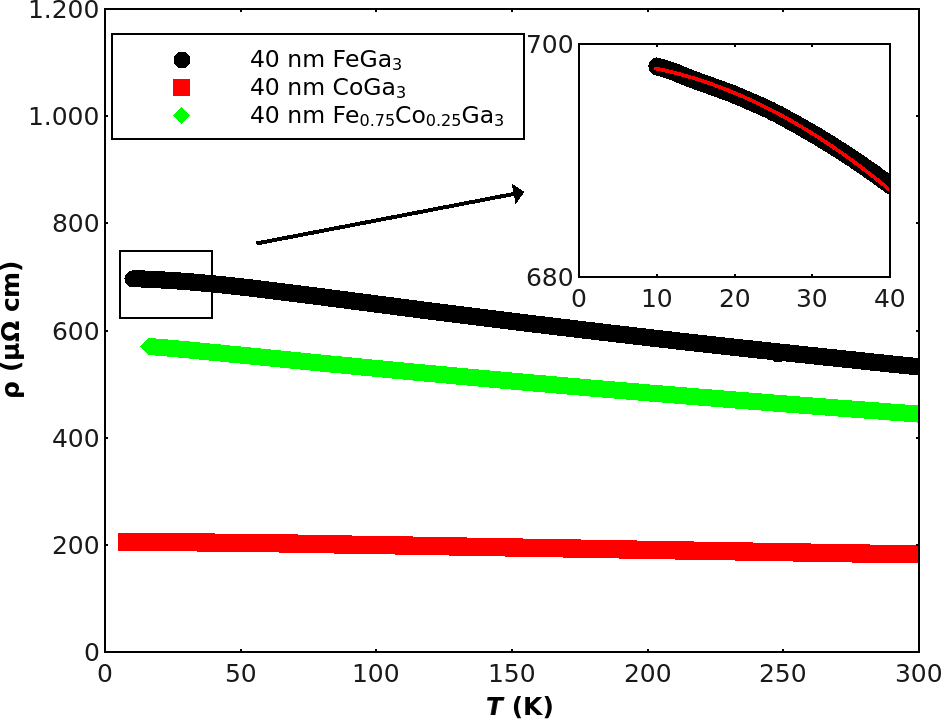
Electrical resistivity of FeGa_3_, CoGa_3_ and Fe_0.75_Co_0.25_Ga_3_ films as a function of temperature. The inset gives a magnified view of the framed FeGa_3_ low temperature behavior together with a fit to ρ = ρ_0_ **+** A*T*^2^ (A = −0.007 K^−2^).

Thus, without explicitly distinguishing between nanocrystalline and amorphous, it is clear that all films are highly disordered with respect to their structure. This immediately poses the question as to how such strong disorder affects electrical transport properties like resistivity, ρ, and Seebeck coefficient, *S*. For amorphous metals, often addressed also as metallic glasses, this question has been analyzed experimentally as well as theoretically for quite some time revealing general trends as well as an improved principal understanding [15–17]. Such a general trend can be expressed by the empirical Mooij’s rule [18] stating that there is a sign change of the temperature-coefficient of resistivity (TCR) of metallic glasses from positive to negative values around a resistivity of 150 µΩ·cm. Thus, around this value, resistivities of metallic glasses are expected to be almost temperature independent. The corresponding experimental data for our present films are presented in Figure 2 for the temperature range 7 K ≤ *T* ≤ 300 K. Three features of these resistivity results are immediately notable: 1) The absolute values for all three films, CoGa_3_, Fe_0.75_Co_0.25_Ga_3_, and FeGa_3_, are extraordinarily high ρ ≥ 200 µΩ·cm. 2) The sequence of these high ρ-values from 200 µΩ·cm for CoGa_3_ to more than 600 µΩ·cm for FeGa_3_ with Fe_0.75_Co_0.25_Ga_3_ in between, but closer to FeGa_3_, reflects the expectation from the corresponding behavior of crystalline samples as mentioned in the introduction: A metallic behavior for CoGa_3_ as opposed to a semiconducting one for FeGa_3_, though with a small band-gap on the order of 0.25 eV [8]. 3) The TCR of the CoGa_3_ films is indeed practically zero while the samples with even higher resistivities exhibit negative TCRs.

All three features may help to distinguish between amorphous/nanocrystalline metallic and semiconducting behavior. Most importantly, in the case of FeGa_3_, a gap of 0.3 eV leads to a resistivity of around 3·10^−3^ Ω·m at 300 K as corroborated experimentally with crystalline bulk samples [8]. This value, however, is larger by a factor of 500 than what is found for our FeGa_3_ films. Furthermore, the observed negative TCR shows a linear temperature-dependence rather than the Arrhenius behavior expected for a semiconductor. Although at low temperatures this may be masked by uncontrolled doping effects. But even in such a case, the pronounced linear temperature-dependence would appear as fortuitous. On the other hand, for the family of high resistivity metallic glasses such a linear behavior is characteristic: A more-or-less linear temperature dependence is observed above about 150 K in all metallic glasses in this family [19]. Even the *T*^2^-behavior ρ = ρ_0_ **+** A*T*^2^ (A < 0) at low temperatures as it is additionally typical for this family of metallic glasses [15] can be found here at T < 40 K (cf. inset of Figure 2). Taken together, the data strongly suggest an interpretation in terms of metallic glasses for all three types of films. In case of FeGa_3_, however, such a conclusion demands that amorphization due to the applied film preparation method results in a higher average density leading to metallic rather than semiconducting properties. The electronic density of states at the Fermi level *N*(*E*_F_) for amorphous FeGa_3_, on the other hand, should be still well below the corresponding value for CoGa_3_ to account for its higher resistivity.

**Figure 3:**
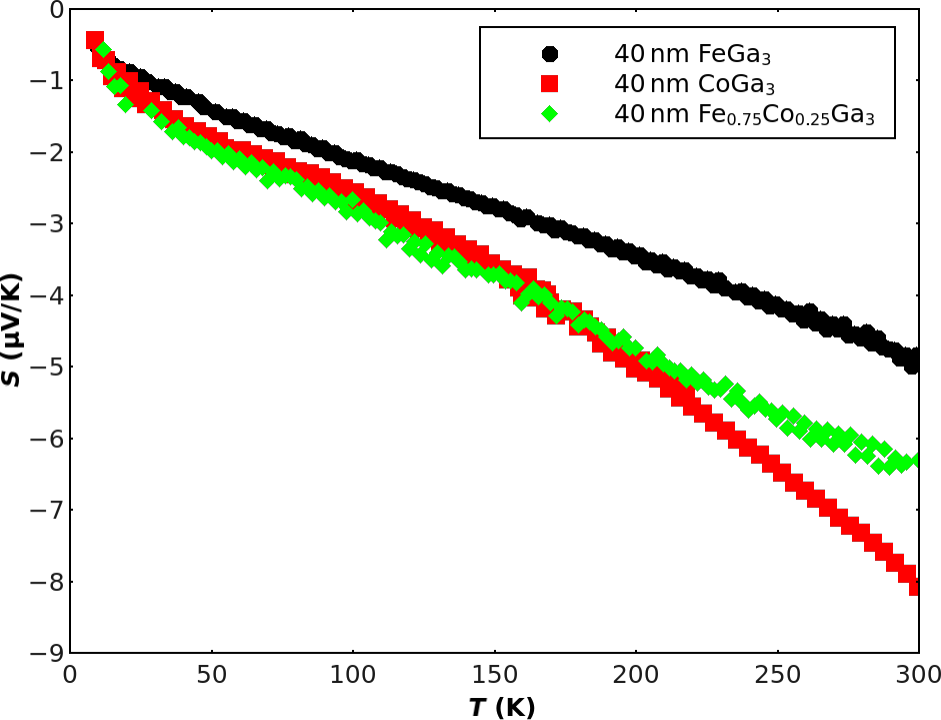
Seebeck coefficients of FeGa_3_, CoGa_3_ and Fe_0,75_Co_0,25_Ga_3_ films deposited on thin glass substrates measured as a function of temperature.

The conclusion on the amorphous state of the presently discussed films has immediate implications on their thermoelectric behavior. First of all, the scattering of electrons is dominated by the static disorder rather than by phonons. As a consequence, phonon drag effects, which usually are responsible for strong non-linear temperature dependence of the Seebeck coefficients *S*(*T*) below typically 100 K in crystalline samples, are expected to be absent. Furthermore, with any ‘sharp’ features in the electronic density of states smeared out by structural disorder, the logarithmic derivative of electric conductivity σ with respect to energy *E* taken at the Fermi energy *E*_F_, (*d*ln σ(*E*)/*d*ln *E*)*_E_*_F_, should also lead to a smooth temperature behavior. Thus, referring to the Mott formula for *S*(*T*) [16],


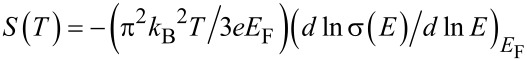


one expects an almost linear *T*-dependence of the second term delivering the sign of *S*(*T*). Indeed, these expectations are mostly confirmed by experiments including metallic glasses containing transition metals with both signs being reported [16,20]. In Figure 3 the *S*(*T*) results for our presently studied films are presented. Again, the data comply with the above expectations for amorphous metals: Smooth, almost linear temperature behavior with no indication for phonon drag peaks in the lower temperature range. Also the magnitude of the *S*(300 K)-values ranging between 4 and 8 μV/K are typical of high-resistance metallic glasses [16]. This clearly confirms the idea of amorphous rather than nanocrystalline structures for the films, especially when comparing these values with corresponding data of crystalline bulk FeGa_3_ samples for which much larger Seebeck coefficients of −350 μV/K [7] or even −563 μV/K [8] at ambient temperature have been reported. Two more details are interesting to note: 1) The negative signs of *S*(*T*) within the observed temperature range of bulk and film samples coincide, indicating a predominant electron transport and 2) according to [20], substituting Fe by a concentration of 5 atom % Co in crystalline bulk samples leads to a transition into a metallic state. Similarly, while 1 atom % Co was found to enhance the magnitude of *S*(300 K) by a factor of two, this enhancement is completely reduced down to the starting value of FeGa_3_ by increasing the Co concentration to either 5 or even 10 atom % [21]. On the other hand, comparison to the present film data shows that at 25 atom % Co the thermoelectric behavior is already very close to that of pure CoGa_3_ supporting the idea of a metallic glass in that case. Unfortunately, *S*(*T*) results for crystalline bulk CoGa_3_ samples are not available to the best of our knowledge, although because of the expected metallic behavior of that system [9] small *S*(300 K)-values of only some μV/K are likely. However, in crystalline samples a possibly present phonon drag may give rise to more pronounced nonlinearities in the temperature dependence of the Seebeck coefficient. Thus, at this point we conclude that the thermoelectric behavior of our films as presented in Figure 3 indicates an electron-dominated transport and that the data are consistent with the assumption of a highly disordered glassy metallic structure.

## Conclusion

Based on a recently developed powder synthesis of FeGa_3_ and CoGa_3_ as well as an intermediate solid solution (Fe_0.75_Co_0.25_)Ga_3_, flash evaporation onto various substrates held at ambient temperature was applied for fabricating the corresponding thin films. This method proved successful in reliably transferring the powder stoichiometry one-to-one into the film. Such a conservation of chemical composition, however, can be obtained only at relatively low deposition temperatures. As a consequence, films of all the above compositions were found to be X-ray amorphous with no indications for the presence of crystallites larger than 5 nm. These new metallic glasses displayed transport properties quite distinct from their crystalline counterparts. The most pronounced difference in this respect is observed for FeGa_3_, which, in its crystalline state, exhibits a semiconducting behavior, though with a small gap of about 0.3 eV. Guided by the performance of standard group-IV semiconductors like Si, which easily can be transformed into an amorphous structure with still semiconducting properties, one would expect amorphous FeGa_3_ to be semiconducting as well. In marked contrast with that expectation, however, one finds in that case the behavior of a typical metallic glass: Much smaller resistivity than what would be expected for a semiconductor with a 0.3 eV gap and a linear rather than exponential temperature-dependence of the resistivity. Correspondingly, the Seebeck coefficient *S*(300 K) is much lower than what is expected for a semiconductor but well within the range typical for metallic glasses. Thus, it appears that for semiconducting intermetallic compounds formed due to the specific hybridization effects between narrow d- and broad sp-bands, rather than due to the formation of strong covalent bonds, structural disorder completely removes the gap. Besides smearing out small features in the electronic density of states, structural disorder may also result in enhanced densities of samples with an accompanying tendency towards the metallic state. As a consequence, the possibility of tuning the electronic properties by substituting Fe by Co in crystalline FeGa_3_ samples and, in this way, shifting the Fermi energy into and out of peaked features in the electronic density of states, is no longer available in the corresponding amorphous films. In the case of CoGa_3_, however, we recently succeeded in transforming an amorphous into a polycrystalline film by annealing at 300 °C for one hour. Comparison to XRD powder data for crystalline CoGa_3_ allowed to identify all significant Bragg peaks (13) in the 2θ-range between 10 and 80° for the annealed sample, although different intensity ratios indicate a preferential growth in the (400)-direction. This recent result not only corroborates the amorphous structure of the as-prepared CoGa_3_ films, but also fosters hope that electronic fine tuning will be possible in future.
